# Two Cases of Infrequent and Simulated Hernia

**Published:** 1829-12

**Authors:** Gilbert T. Burnett

**Affiliations:** Surgeon, &c.


### HERNIA.

*Two Cases of infrequent and simulated Hernia.* Communicated by GILBERT T. BURNETT, Esq. Surgeon, &c.

T. P., aged sixty, entered on a detail of his complaints by asking whether it were "possible for the bladder to escape from the belly into the purse? for," continued he, "I have been for several years subject to an enlargement in my left groin, which, within the last twelve or eighteen months, has considerably increased; and it does seem to me that, whenever I make water, the swelling diminishes in size." The question was one not difficult of solution, and the history of the case appeared to be shortly this: Being, from his occupation, (a master builder,) obliged to mount ladders, and trust himself frequently on places of doubtful footing, he had often had falls of greater or less severity, and to a strain, or some such accident, the origin of the tumor might be traced. The exact period of its duration he knew not, but thought it had probably been unobserved or only half-noticed for a considerable time; for, even after his attention was more particularly drawn towards a soft lump in his right groin, which disappeared under the pressure of the finger, it was not until it became larger than a walnut, and constantly returned shortly after the finger was removed, that he became at all uneasy about it. He formerly consulted a surgeon of some eminence in this town, who laughed at his idea of the size of the tumor being lessened by the passage of urine; told him that it was an ordinary rupture, and ordered him to wear a common truss. This he attempted to do; but, after trying various instruments, and several modifications of pads and springs, the intolerable pain which the pressure gave, and the constant reappearance of the swelling below the cushion, compelled him shortly to desist; and as the tumor itself, when unmolested, gave him no pain, he allowed two years more to elapse without any further surgical treatment being had recourse to. During this period no fresh symptoms made their appearance; but the still-increasing magnitude of the tumor distressed him, and hence he was led to consult another surgeon, who, after two examinations, decided it to be formed by the protrusion of a part of the bladder, and dissuaded him from the use of any truss, as it had previously occasioned so much uneasiness. These conflicting opinions and directions, though probably both justified by the different stages of the disease at such distant periods, distracted his mind; and hence the question he proposed as to the possibility of the bladder protruding through the abdominal parietes and entering the scrotum.

When I examined him, the right inguinal canal was distended in the greater part of its course; but, as is frequently the case in old ruptures, the internal ring seemed nearer than it should be to the external, although the course of the hernia did not lead to the supposition that it had been originally direct. The tumor occupied the right side of the scrotum nearly to the bottom, and behind and under it the testicle might be indistinctly felt. Had not his questions raised a suspicion of the nature of this case, the fluctuation of the tumor and its situation might have given the idea of its being hydrocele of the cord; but its clearly decreasing in size on pressure, which pressure forced the urine to be passed through the ordinary canal, would have decidedly cleared up any ambiguity which the first examination might have perchance excited.

As at this period (March 1826,) it had so long existed, probably for five years or upwards; as the protruded portion of the bladder seemed to have formed adhesions with the surrounding cellular membrane, for, although the contents of this cystic pouch could be evacuated by pressure, the pouch itself was irreducible; as the patient's health was in general good, the bowels regularly open, and no suspicion of any portion of intestine being implicated in the descent; as it was rather an inconvenient than a painful tumor, there seemed nothing feasible to be recommended save a suspensory bandage, to check its further increase, and caution to avoid, by any sudden or violent exertion, the complicating a cystic with an intestinal rupture. Adhesions had probably early formed, even before the first consultation; and hence, although the hernia was apparently reduced, the fluid only was returned, and the sac was subject to the pressure of the truss, giving rise to the pain alluded to, and rendering it impracticable to pursue the plan his first surgeon recommended.

This patient lived three years after the period now referred to, with no further inconvenience than being obliged to use his hand as a detrusor urinæ when he wished entirely to evacuate his bladder. Indeed, he died only last spring, and from inflammation of the lungs. I had always kept my eye upon him, and did anxiously desire to have had an opportunity of examining the parts; but prejudice, and a mistaken respect for the dead, (which is rather an insult and a crime to the living,) prevented that which would have rendered this case still more interesting and satisfactory, by perfecting the record.

The above is the only case of cystocele that I have ever met with, or indeed been able to get any contemporary account of, among my professional acquaintances. It is, indeed, I believe, a very rare disease; and, although there are several cases on record before this occurred to me, I had always regarded it as one of those possibilities mentioned in nosologic works for the sake of system, rather than as a probability of so much practical importance. In having presumed to think these details might not be unworthy a page in the London Medical and Physical Journal, I have been influenced by the consideration of how much discredit, perhaps unjustifiably, attaches to a surgeon who, forgetting those rare possibilities, ventures a decided and too hasty diagnosis, which is subsequently disputed, and ultimately proves to be incorrect; and patients seldom acknowledge that they have had any previous consultation until they have obtained the opinion sought, and then immediately turn round with "But Mr. A., B., or C. says so and so."

Mr. Pott, in his surgical works, mentions two cases of cystic hernia, which he observes are the only two he ever met with; and, as more advantage is often derived from recalling to public attention the experience of former times, than in relating the more limited practice of our own, it may not be perhaps irrelevant shortly to advert to them.

"A poor fellow," writes Mr. P. "who worked with a farmer at Islington, came to St. Bartholomew's, with a large troublesome swelling in his scrotum. The tumor was large, tense, of a pyriform figure, palpably contained a fluid, gave no pain, but from its weight when full; and had every mark of a hydrocele, except that the testicle was perfectly distinguishable at its bottom. While I was hesitating concerning this circumstance, the man said, 'Sir, I can get rid of it all by pissing, but it fills again in a few hours, especially if I drink. Upon my seeming to disbelieve what he said, he took up his scrotum, and, squeezing it together with some violence, discharged the whole by the urethra."

The other case, which is one of considerable interest, occurred in a boy six years old, who at that age was seized with an acute pain about the region of the pubes, which lasted for an hour and a half, and then suddenly ceased. After a few days a small tumor was discovered in the groin, about the size of a pea; but, as it gave no pain, no notice was taken of it. After a time it increased in size, got lower and lower into the scrotum, and he found himself frequently urged to make water. He was examined by a practitioner in his neighbourhood, who, not knowing what to make of it, advised the letting it alone. Others subsequently deemed it a scirrhous testicle, and proposed castration. Mr. P. was not consulted till the boy was thirteen years old: he did not think it was a scirrhous testicle, yet only one testicle could be felt. The tumor was perfectly even on its surface, indolent, had a stony incompressible kind of hardness, but never occasioned pain in the back and loins. The trouble it gave the lad, and its disposition to increase, seemed to authorize its removal, and the operation was performed. A firm white membranous cyst, connected loosely with the cellular membrane, in the same manner as a hernial sac, was dissected all round, and found to be dependent from a membranous neck or duct, which passed through the opening of the abdominal muscles; which neck was cut through immediately above the tumor, upon the division of which, about four ounces of clear liquid issued, and, the mouth of the cyst expanding, disclosed a stone exactly resembling what is found in the human bladder. Subsequently the lad passed urine through the wound, and the case, which ultimately did well, proved to have been one of stone complicated with cystocele.

I purposely avoid referring to any of the other varieties of cystic hernia, and have merely quoted the above cases to prove how difficult is the diagnosis where it may be of extreme importance to distinguish rightly. In Manuals of Surgery, the characters of each form are often so laid down, that a novice might think it scarcely possible to err; but those who have practically attended to the matter well know that the most familiar and common ruptures are occasionally ambiguous and obscure.

Allow me to mention a case of a different kind to the foregoing, but yet which, I think, is immediately in point.

I was sent for to attend J. C., aged nineteen, who, while lifting a heavy burden, was suddenly seized with a pain in the right groin, and, on putting his hand to the part, perceived a lump about the size of a nutmeg, which was incompressible, and which resisted his attempts to return it into the abdomen, whence it had, during violent exertion, descended. On inquiry, I ascertained that he had long been occasionally subject to a small swelling in the part, but which had never before protruded so far, had always been without pain, and which in general receded on slight pressure, or when he was in a recumbent posture. It had now, however, been down for three days, and had resisted every effort at reduction. The first two days it had given him but little uneasiness, but for several hours past it had been getting more and more painful; and I was therefore sent for to examine it. It was a small tumor within the inguinal canal, nearer to the internal than to the external ring, but slightly compressible, and painful to the touch. There was much anxiety and irritability about him, the pulse quick, and the bowels had not been open since the last descent of this inguinal tumor. I decided immediately in my own mind that it was a case of bubonocele, bled him freely, employed the taxis, yet unsuccessfully, the tumor resisting all attempts to return it into the abdomen, whence it had evidently emerged. It had a less elastic feel than ordinary intestinal hernia, being in fact more like what might be supposed to indicate the presence of a lump of half-hardened feces in the pouch. A hot bath was then ordered, in which the patient was put, when, on renewing the taxis, and taking hold of the scrotum, there was in it but one testicle, and that on the left side; the right side of the scrotum had no testicle within it. A new light was thus thrown on the case, which proved to be one of those infrequent and tardy descents of the testicle, which, from its sudden appearance on exertion, its irreducibility, and the peculiar coincidence of constipation, had in some measure simulated an intestinal hernia. The means resorted to had so far relaxed the parts, that the testicle was subsequently returned, and an operation, which I had contemplated, escaped.

Such a case as this might mislead once, but such an event could never occur a second time: its notice may, perhaps, prevent even such a solitary error.

Malingerers not unfrequently simulate hernia by forcing up one or both of their testicles into the groins; and a case has been mentioned in which a soldier, who wished to gain his discharge, thus counterfeited a double rupture. In this man the cremaster muscles were so powerful, and so directly under the control of the will, that he was able, by their action only, to elevate the testes to the groins, and keep them suspended at the external rings.

A somewhat analogous instance is on record in a female servant, strong, stout, and in good health, who had a painful tumor in each groin, which had clearly passed out of the abdomen, but which resisted every attempt at their reduction. They subsequently became so painful as to hinder her working, and were accordingly removed by operation. They were then seen to be the ovaria, which had thus descended to the groins. Previous to their excision, she was plump, had large breasts, and menstruated freely: afterwards, although she enjoyed good health, she became thinner and more muscular, her breasts disappeared, and she never menstruated.

Let me, in concluding, mention two other cases of hernia, which, although not interesting from their ambiguity, are warnings of the extent which neglected bubonocele will reach, and which, I am fain to believe, are not frequent in their occurrence. I have never seen but two, one in hospital, the other in private practice. The first died, and was examined; the latter, which is of less extent, is in a man now living, though in a pitiable state. In both these persons, the whole or the greater part of the intestines and omentum were extra-abdominal; for, from dislike to wearing a truss in the early stages, a mere bubonocele had become an adherent scrotal hernia, and gradually increased to such a degree that the scrotum formed a pouch reaching to the knees, the integuments of the abdomen being greatly dragged down; the penis entirely buried in the tumor; and the urine passed out at an aperture at the upper periphery of these immense oschroceles. The first was a good-tempered old man, who thought less of his enormous purse, than of the gout, with which he likewise was afflicted; but the other, a much younger man, though less burdened, seems but little familiarised to its weight and the chafing, which is a great impediment to walking, and far from reconciled to the impotency which has ensued; for the increase of the tumor having entirely enveloped the penis, although tortured by sexual desires, he has been absolutely emasculated for years.

